# Supplementary material for: Clinical Impact of Sarcopenia and Inflammatory/Nutritional Markers in Patients with Unresectable Metastatic Urothelial Carcinoma Treated with Pembrolizumab
Source: Diagnostics (Basel). 2020 May 15;10(5):310. doi: 10.3390/diagnostics10050310 (PMC7277993; doi:10.3390/diagnostics10050310)
Supplement: Supplementary file 1 [file diagnostics-10-00310-s001.zip › Supplementaly Files/Supplementary Table 1.docx]

| **Table S1.** CONUT score calculation formula | | | | |
| --- | --- | --- | --- | --- |
| **Variables** | **Degree of malnutrition** | | | |
|  | **None** | **Mild** | **Moderate** | **Severe** |
| **Serum albumin (g/dL)** | ≥ 3.5 | 3.00 - 3.49 | 2.50 - 2.99 | < 2.5 |
| **Score** | 0 | 2 | 4 | 6 |
| **Total lymphocyte count (/mm^3^)** | ≥ 1600 | 1200 - 1599 | 800 - 1199 | < 800 |
| **Score** | 0 | 1 | 2 | 3 |
| **Total cholesterol (mg/dL)** | ≥ 180 | 140 - 179 | 100 - 139 | < 100 |
| **Score** | 0 | 1 | 2 | 3 |
