# Supplementary material for: Clinical Impact of Sarcopenia and Inflammatory/Nutritional Markers in Patients with Unresectable Metastatic Urothelial Carcinoma Treated with Pembrolizumab
Source: Diagnostics (Basel). 2020 May 15;10(5):310. doi: 10.3390/diagnostics10050310 (PMC7277993; doi:10.3390/diagnostics10050310)
Supplement: Supplementary file 1 [file diagnostics-10-00310-s001.zip › Supplementaly Files/Supplementary Table 2.docx]

| **Table S2a.** Adverse events and immune-related adverse events | | | |
| --- | --- | --- | --- |
| **Adverse event** | **Any grade, n (%)** | **Grade1-2, n (%)** | **Grade3-4, n (%)** |
| Any adverse event | 12 (44%) | 7 (26%) | 5 (19%) |
| Any immune-related event | 10 (37%) | 5 (19%) | 5 (19%) |
| **General adverse event** | | | |
| Fatigue | 10 (37%) | 10 (37%) | 0 |
| Nausea | 2 (7%) | 2 (7%) | 0 |
| Anemia | 1 (4%) | 1 (4%) | 0 |
| **Immune-related adverse event** | | | |
| interstitial pneumonia | 4 (15%) | 1 (4%) | 3 (11%) |
| dermatopathy | 3 (11%) | 3 (11%) | 0 |
| abnormal hepatic function | 2 (7%) | 1 (4%) | 1 (4%) |
| Hypothyroidism | 1 (4%) | 1 (4%) | 0 |
| Type 1 diabetes mellitus | 1 (4%) | 0 | 1 (4%) |

| **Table S2b. impact of sarcopenia on irAE (G3 or higher)** | | | | |
| --- | --- | --- | --- | --- |
|  | | **Univariate** | | |
|  |  | **HR** | **95% CI** | **P value** |
| Sarcopenia | No | 1 | 0.02-1.46 | *0.1* |
|  | Yes | 0.15 |  |  |
| irAE = immune-related adverse event; HR = hazard ratio; CI = confidence interval | | | | |

| **Table S2c. Association of sarcopenia and irAE** | | | | | |
| --- | --- | --- | --- | --- | --- |
|  |  | **Total** | **No sarcopenia** | **Sarcopenia** | **P value** |
| irAE | < G3 | 22 (81%) | 9 (75%) | 13 (87%) | 0.63 |
|  | ≥ G3 | 5 (19%) | 3 (25%) | 2 (13%) |  |
| irAE = immune-related adverse event | | | | | |
| Fisher’s exact test | | | | | |
