# Supplementary material for: Clinical Impact of Sarcopenia and Inflammatory/Nutritional Markers in Patients with Unresectable Metastatic Urothelial Carcinoma Treated with Pembrolizumab
Source: Diagnostics (Basel). 2020 May 15;10(5):310. doi: 10.3390/diagnostics10050310 (PMC7277993; doi:10.3390/diagnostics10050310)
Supplement: Supplementary file 1 [file diagnostics-10-00310-s001.zip › Supplementaly Files/Supplementary Table 3.docx]

| **Table S3.** Univariate analysis for correlation between PFS/OS and  changes of CRP 3M after the first Pembrolizumab | | | | | | | | | |
| --- | --- | --- | --- | --- | --- | --- | --- | --- | --- |
| **Variable** |  | **PFS** | | |  | **OS** | | |  |
|  |  | **Univariate** | | |  | **univariate** | | |  |
|  |  | **HR** | **95% CI** | ***P* value** |  | **HR** | **95% CI** | ***P* value** |  |
| CRP change  3M | Low→Low High→Low Low→High→Low | 1 | 7,67-118 | *<0.0001* |  | 1 | 7.98-101.2 | *<0.0001* |  |
|  | the others | 30.15 |  |  |  | 28.4 |  |  |  |
| HR = hazard ratio; CI = confidence interval; irAE = immune-related adverse events;  CRP = C-reactive protein; High = CRP≥0.5; Low = CRP<0.5 | | | | | | | | | |
