# Supplementary figures and images for: Clinical Impact of Sarcopenia and Inflammatory/Nutritional Markers in Patients with Unresectable Metastatic Urothelial Carcinoma Treated with Pembrolizumab
Source: Diagnostics (Basel). 2020 May 15;10(5):310. doi: 10.3390/diagnostics10050310 (PMC7277993; doi:10.3390/diagnostics10050310)

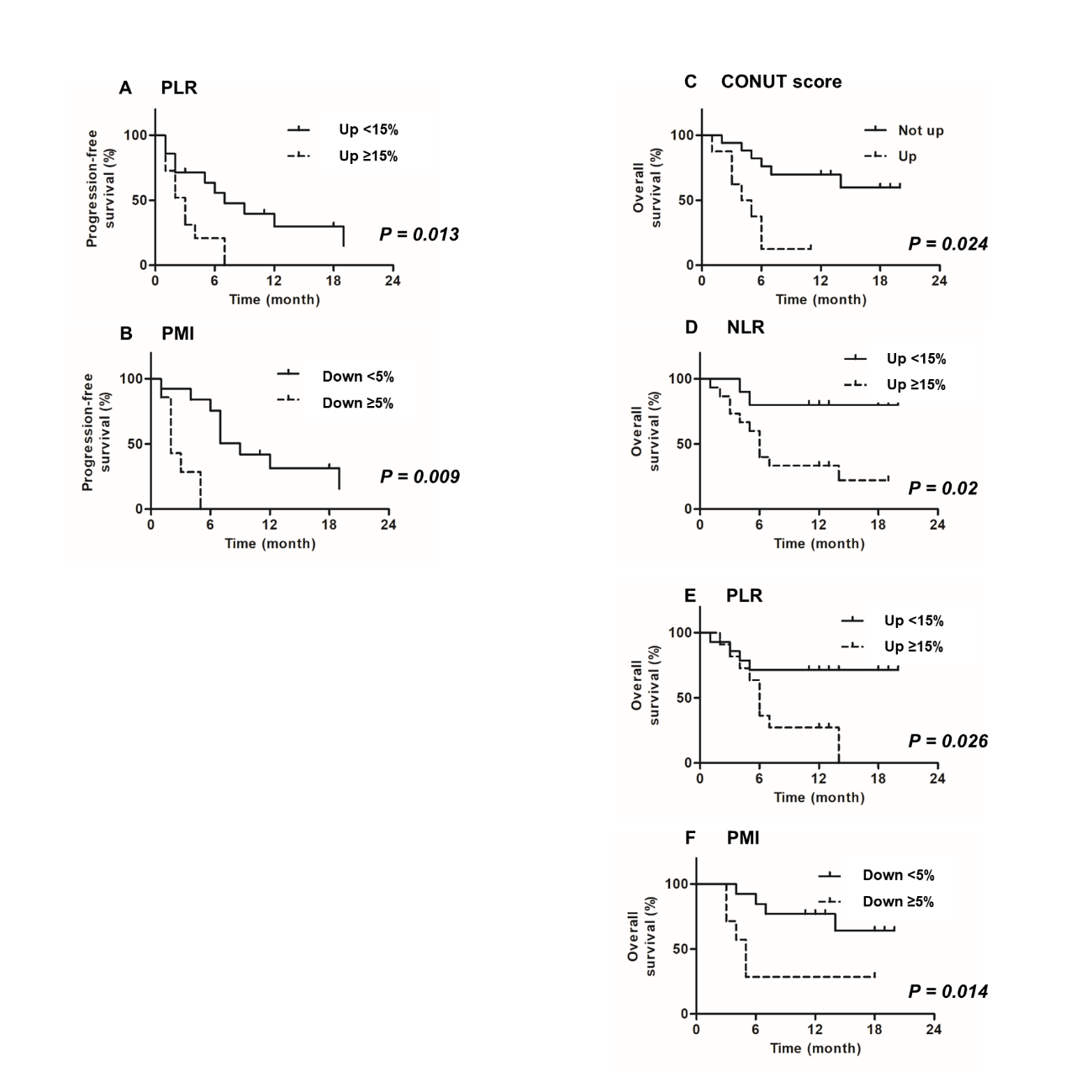

Supplement: Supplementary file 1 [file diagnostics-10-00310-s001.zip › Supplementaly Files/Supplementary Figure 1.docx]
